# Whose helping hurts? Source and construct differences in unhelpful workplace social support

**DOI:** 10.3389/fpsyg.2026.1811297

**Published:** 2026-05-13

**Authors:** Anne Jeannette Ponsteen, Kristoffer Holm

**Affiliations:** 1 Department of Urban Studies, Malmö University, Malmö, Sweden; 2 Department of Urban Studies, Malmö University, Center for Work Life Studies, Malmö, Sweden

**Keywords:** dysfunctional social support, organizational-based self-esteem, stress-as-offence to-self theory, turnover intentions, unhelpful workplace social support

## Abstract

**Introduction:**

Although workplace social support is typically viewed as a beneficial job resource, it can also function as an interpersonal stressor when perceived as unhelpful or subtly devaluing. This study aims to advance understanding of how unhelpful support operates at the workplace by studying three aspects of the construct: (1) providing the first empirical comparison between unhelpful workplace social support (UWSS) and dysfunctional social support (DSS), (2) examining whether unhelpful workplace social support (UWSS) can be meaningfully distinguished by source (coworkers vs. supervisors), and (3) testing organization-based self-esteem (OBSE) as a mechanism linking these forms of unhelpful help to negative employee outcomes, including the novel outcome of turnover intentions.

**Methods:**

Survey data from 157 working adults were analyzed using factor- and mediation analyses.

**Results:**

Results indicate that dysfunctional social support is empirically distinct from unhelpful workplace social support and emerges as a slightly stronger predictor of organizational frustration, counterproductive work behavior, and turnover intentions. Significant indirect associations *via* OBSE were only observed for the relationships between partial supervisor unhelpful workplace social support and turnover intentions, as well as (partially) for the relationship between dysfunctional social support and turnover intentions. Dysfunctional social support showed direct relationships with organizational frustration, counterproductive work behavior, and turnover intentions, and demonstrated more consistent relationships in our model.

**Discussion:**

These findings underscore the impact of subtle, low-threshold, normalized forms of unhelpful support for organizations.

## 1 Introduction

Social support at work is generally considered a job resource that helps employees cope with stress and enhances well-being (for a comprehensive review, see Jolly et al., 2020). However, a growing body of research highlights that not all workplace social support is beneficial; it can also have detrimental effects when perceived as unhelpful or even harmful (Deelstra et al., 2003; Beehr et al., 2010; Deelstra, 2003). Based on this notion, Gray (2018) introduced a new construct: *unhelpful workplace social support* (UWSS), which refers to any action taken by a colleague or supervisor that is intended to benefit another employee but is perceived as unhelpful or even harmful by the recipient (Gray, 2018, p. 2). The emphasis on good intent despite negative outcomes differentiates UWSS from related concepts like workplace incivility (e.g., Andersson and Pearson, 1999) or interpersonal conflict (Spector and Bruk-Lee, 2008), which involve malicious or ambiguous intent, or a complete absence of resource provision (Hughes et al., 2022). Some findings suggest the costs associated with unhelpful support may outweigh the benefits of helpful support at times (Gray, 2018). Rather than alleviating strain, UWSS can thus act as a job demand or interpersonal stressor, triggering a variety of strain outcomes. Empirical studies have linked UWSS to higher levels of job-related negative affect, organizational frustration, emotional exhaustion, burnout, physical symptoms, lower competence-based self- esteem, counterproductive work behaviors, and reduced coworker satisfaction (Gray, 2018; Gray et al., 2025; Hughes et al., 2022; Hughes et al., 2024). In addition to UWSS, Semmer et al. (2025) introduced dysfunctional social support (DSS), a related construct that captures subtle, low-threshold forms of support that are likely to be perceived as unhelpful and/or harmful. Although research on these less positive forms of support has begun to emerge, further research is required to clarify the mechanisms through which UWSS and DSS operate, how they relate to each other, and to determine why and when their effects become harmful.

To date, UWSS research has predominantly examined coworker interactions, overlooking the possibility that supervisors can also engage in unhelpful supportive behaviors. Given their position of authority, and the expectation to support their team members, supervisor’s support efforts may be especially influential on employees’ perceptions, behaviors, and well-being. A recent conceptual extension in UWSS literature, unhelpful supportive leadership (Gray, 2021), suggest that UWSS provided by supervisors might differ from UWSS from coworkers in both form and outcome. Indeed, a comparison between unhelpful supportive leadership (USL) and more traditional negative leadership styles found USL to be more detrimental to psychological need fulfillment, such as perceived competence and relatedness (Gray, 2021). Supporting this, preliminary studies on similar negative social support concepts indicate that the source of support matters, finding stronger negative effects of supervisor support on strain compared to colleague support (Beehr et al., 2010). Although directly comparing supervisor and coworker ratings may be challenging, since coworker ratings refer to coworkers in general whereas supervisor ratings concern a single, clearly identifiable individual, examining the impact of each source can provide valuable insights into the potential importance of the support provider, given that coworkers and supervisors occupy very different roles for the recipient. For example, the heightened impact of supervisor support likely stems from employees’ reliance on supervisors for performance evaluations and career advancement (Deelstra et al., 2003; Beehr et al., 2010). However, these streams of research have not yet been fully integrated into the UWSS framework, as no study has systematically compared coworker versus supervisor UWSS utilizing the full UWSS measurement scale. As a result, it remains unclear whether unhelpful support from these different sources share underlying psychological mechanisms or operates through distinct pathways.

Building on these gaps, the present study pursues three aims. Specifically, we seek to examine whether UWSS and dysfunctional social support (DSS) represent conceptually and empirically distinct constructs. In addition, we investigate whether employees perceive and receive UWSS differently from coworkers versus supervisors, thereby addressing the role of the support source in shaping UWSS experiences and consequences. Finally, we test organization-based self-esteem (OBSE) as a mediator to link UWSS and DSS to relevant outcomes, such as organizational frustration (OF), counterproductive work behavior (CWB), and turnover intentions (TI).

**Figure 1:**
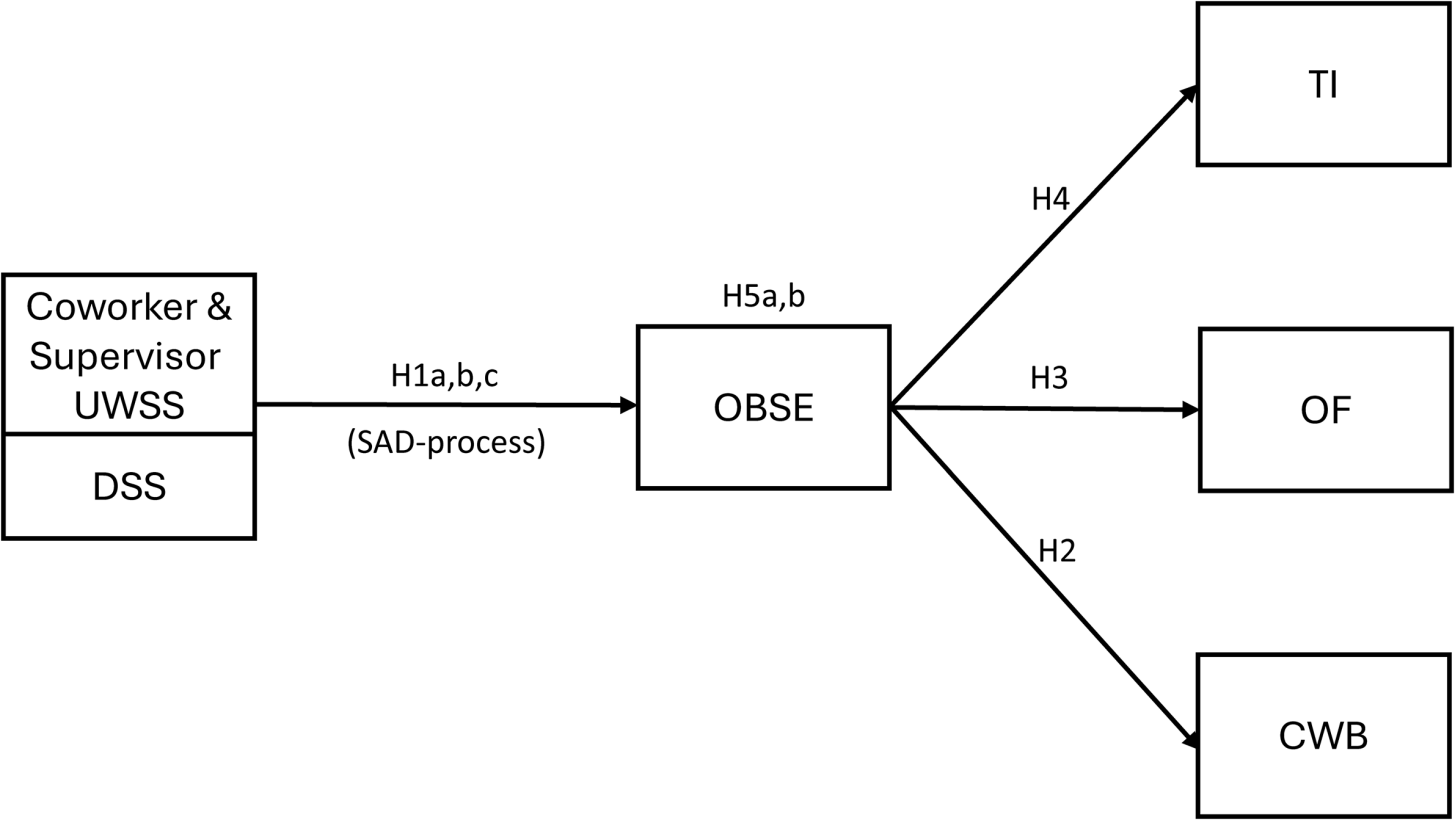
Hypothetical model and the study hypotheses.

To integrate these aims, we present and test a model in which OBSE is proposed as a potential mediator between UWSS/DSS and outcomes, including OF, CWB, and TI. In the present study, we primarily draw on the Stress as Offence to Self-theory (SOS; Semmer et al., 2019), and present a hypothesized mediation model of UWSS outcomes, see Figure 1.

## 2 Theoretical framework and hypotheses development

Earlier research has explored potential negative effects of social support by studying the reverse-buffering effects of social support at work, i.e., when social support exacerbates rather than buffers the relationship between stressor and strain outcomes (Jolly et al., 2020; Kaufmann and Beehr, 1986). While negative outcomes of social support have primarily been explained through recipient or provider characteristics, Gray’s (2018) concept of unhelpful workplace social support introduces a new perspective. UWSS locates the explanation for negative outcomes within the supportive act itself, arguing the nature of social support, specifically the way support is provided, determines whether it is perceived as helpful or harmful. Within the UWSS framework, the UWSS-measure distinguishes six forms of unhelpful support: critical, imposing, partial, undependable, short-sighted, and uncomforting social support (see Gray (2018) p. 62 for a categorization of UWSS types with examples). Additionally, poorly assigned social support can be included as a seventh type, though it applies only to supervisor-provided support (Gray, 2018). These types are suggested to be interrelated but distinct, each offering unique explanatory value and differing in their outcomes and level of impact. For example, Hughes et al. (2024) identified *partial* and *undependable* support as the strongest predictors in their multi-theory model. Similarly, using dominance analyses, Hughes et al. (2022) found UWSS types differentially predict strain outcomes: *critical social support* was the strongest predictor of abuse and production deviance (facets of counterproductive work behaviors), *conflicting social support* most strongly predicted emotional exhaustion, and *undependable social support* was the key predictor of withdrawal behaviors. Although the UWSS types are theoretically distinguished, the UWSS research field is still relatively young, and further research is needed to establish their distinctiveness.

### 2.1 Clarifying conceptual distinctions and differentiating support sources

Parallel to the emergence of research on UWSS, Semmer et al. (2025) developed a new but related concept and measurement scale called dysfunctional social support (DSS). Like UWSS, DSS covers supportive behaviors that are (likely) to be perceived as derogatory and not sufficiently appreciative, but it distinguishes itself by its focus on subtility. Unlike UWSS, DSS includes lower-threshold behaviors and more subtle threats that reflect indirect ways of failing to convey a message of care, esteem, and appreciation (Semmer et al., 2025). DSS avoids strong negative statements that are included in the UWSS scale, as the authors believe stressors (stemming from unhelpful support) can be far more subtle than what the UWSS scale allows to measure (Semmer et al., 2025). Semmer et al. (2025) found DSS to explain incremental variance in strain outcomes such as irritation, somatic complaints, job satisfaction, and attitudes towards life, but not intention to quit. Moreover, Semmer et al. (2025) suggest that both UWSS and DSS could be employed simultaneously to assess their relative importance. However, no study has yet established whether these two constructs are empirically distinguishable from each other, or rather capture overlapping aspects of unhelpful support. Addressing this represents a central contribution of the present study, as clarifying the relationship between UWSS and DSS is essential for advancing theory on unhelpful support. Consequently, in this study, we will measure both constructs and conduct an exploratory analysis to test whether or not UWSS and DSS have distinct explanatory value and relate differently to the other variables tested in our model. In doing so, we also assess whether DSS, with its focus on subtle and low-threshold behaviors, provides additional explanatory value beyond UWSS, which covers more overt unhelpful acts of support.

Moreover, social support at work can originate from different actors. Existing UWSS literature has either focused on support received from direct colleagues or has not differentiated between coworker and supervisor support (e.g., Gray, 2018; Hughes et al., 2022; Hughes et al., 2024). Within our knowledge, just two papers studied UWSS stemming from supervisors (Gray, 2021; Gray et al., 2022). In these studies, unhelpful supportive leadership (USL) was defined as “leaders who perform supportive acts that the recipient believes were intended to benefit them but are perceived as unhelpful or harmful” (Gray, 2021, p. 1). USL is grounded in UWSS literature and employs a shortened and adapted version of the UWSS measurement scale. However, two limitations of these studies include not being able to distinguish between the different UWSS types as the USL scale measured UWSS as one single construct. Additionally, both studies only measured unhelpful leadership support, precluding a comparison between unhelpful support from supervisor vs. co-workers (Gray, 2021; Gray et al., 2022). The current study addresses this issue by measuring UWSS experiences from both co-workers and supervisors, utilizing the full UWSS-scale so that the two sources can be adequately compared.

### 2.2 Integrating stress-as-offence-to-self theory and hypotheses development

The stress-as-offence-to-self (SOS) theory aims to explain why people react the way they do to external cues. The theory assumes that achieving, maintaining, and protecting a positive self-view is a basic human need people constantly try to fulfill or uphold (Semmer et al., 2019). The SOS model posits that (subtle) threats to an individual’s personal or social self- esteem resulting from interpersonal stressors can engender physiological, behavioral, and psychological strain (Semmer et al., 2019; Hughes et al., 2022). In line with this, UWSS/DSS as interpersonal stressors likely act as a threat to the recipient’s self-esteem (Gray et al., 2025; Hughes et al., 2022). SOS makes a distinction between personal and social self-esteem. Social self-esteem refers to the degree to which one feels esteemed, acknowledged, and appreciated by significant others, thus it strongly depends on one’s social surroundings. When these social surroundings elicit experiences that induce feeling derogated, ignored, excluded, or treated unfairly, the social self-esteem is threatened through the Stress-as-Disrespect (SAD) process (Semmer et al., 2019).

By drawing on the SOS framework, Hughes et al. (2022) linked UWSS to strain outcomes (CWBs and job-related negative affect). Their findings suggest UWSS can be placed within the SOS-model as an interpersonal stressor that threatens personal or social self-esteem, which engenders stress that can be vented by showing behavioral (CWB), physiological, and psychological strain (Hughes et al., 2022). Semmer et al. (2025) also expand on the SOS-model to explain how DSS interactions threaten the self. However, both studies do not directly examine the relation between UWSS/DSS and self-esteem. Therefore, it is still unknown if there is a direct relationship between UWSS/DSS and outcomes, or if this effect is (partially) mediated by self-esteem. Organization-based self-esteem (OBSE) has been defined as “the degree to which organizational members believe that they can satisfy their needs by participating in roles within the context of an organization” (Pierce et al., 1989, p. 625). OBSE could be considered a way of defining social-self-esteem specific to organizational settings, as it is a form of social self-evaluation reflecting one’s self-perceived value as an organization member, and one’s acceptance within said organization. However, it should be noted that OBSE is a context-specific operationalization of social self-esteem, aligned with the current proposed model, but it might not capture the full range of processes related to self-esteem as implied in the SOS theory.

Drawing on the SOS model (Semmer et al., 2019), we argue that UWSS/DSS experiences might threaten employees’ organization-based self-esteem (OBSE) through the Stress-as- Disrespect process. In turn, OBSE is proposed as a potential link that connects UWSS/DSS to various outcomes, including organizational frustration (OF), counterproductive work behavior (CWB), and turnover intentions (TI). The association between UWSS and turnover intentions has not yet been studied. However, low OBSE has been associated with withdrawal behaviors (Carson et al., 1997). Threats to OBSE are likely to undermine employees’ sense of belonging, because OBSE reflects their self-perceived value and acceptance within the organization, suggesting OBSE may be particularly relevant for explaining withdrawal-related outcomes in UWSS/DSS models. Including turnover intention as an outcome allows us to examine whether the effects of UWSS extend beyond strain reactions to more far-reaching outcomes, thereby providing a more complete understanding of the organizational implications of UWSS.

Taken together, the following hypotheses reflect the three interrelated components under study of how unhelpful support operates in the workplace, with a primary focus on distinguishing between UWSS and DSS and their impact on employee outcomes. In addition, the hypotheses address whether these associations differ by support source (coworkers vs. supervisors) and explore organization-based self-esteem (OBSE) as a potential underlying mechanism. Consecutively, we hypothesize the following:

*H1a*: Unhelpful workplace social support from co-workers will be negatively associated with organizational-based self-esteem.

*H1b*: Unhelpful workplace social support from supervisors will be negatively associated with organizational-based self-esteem.

*H1c*: Dysfunctional social support will be negatively associated with organizational-based self- esteem.

*H2*: Lower levels of organizational-based self-esteem will be associated with higher levels of counterproductive work behaviors.

*H3*: Lower levels of organizational-based self-esteem will be associated with higher levels of organizational frustration.

*H4*: Lower levels of organizational-based self-esteem will be associated with higher levels of turnover intention.

*H5a*: Unhelpful workplace social support will be indirectly associated with negative employee outcomes (counterproductive work behaviors, organizational frustration, and turnover intention) *via* organizational-based self-esteem.

*H5b*: Dysfunctional social support will be indirectly associated with negative employee outcomes (counterproductive work behaviors, organizational frustration, and turnover intention) *via* organizational-based self-esteem.

## 3 Methods

### 3.1 Participants, procedure, and ethical considerations

The population of interest included working adults aged 18 years or older. Participants were recruited using a combination of convenience and snowball sampling methods. Recruitment primarily took place through the researchers’ personal networks, supplemented by on-site sampling around a university campus in southern Sweden. Individuals who agreed to participate were directed to complete an online survey. The sample consisted of 161 participants, of which four participants were removed because they did not meet the inclusion criteria, resulting in a final sample size of 157 (57 males, 98 females, 2 non-binary) with a mean age of 33.73 (*SD* = 12.75), mean length of employment of 5.06 years (*SD* = 7.45), and mean weekly working hours of 30.85 (*SD* = 14.20). Of the sample, 36 (22.9%) reported they had a supervisor position with staff responsibilities, 34 (21.7%) reported they had a supervisor position without staff responsibilities, and 87 (55.4%) had no supervisor position.

Before starting the survey, participants received written information about the study’s purpose, types of questions included, and the estimated completion time. The survey’s front page explained participation was entirely voluntary and that participants could withdraw at any time without consequences. It further described how personal data would be processed and stored in accordance with data protection regulations. No sensitive personal data was collected, and responses were anonymous. Participants provided informed consent by selecting “Yes” before proceeding. Formal ethical approval was not required for this research, as the study involved minimal risk, gathered no sensitive personal information, and relied exclusively on anonymous questionnaire data.

### 3.2 Measures

#### 3.2.1 Demographics

Demographic questions included in the survey concerned gender, age, country of residence, occupational title, length of employment (tenure), average weekly working hours, and supervisor position.

#### 3.2.2 Unhelpful workplace social support (UWSS)

Unhelpful workplace social support was measured using the UWSS scale developed by Gray et al. (2019). The scale consists of 24 items, with four items measuring each UWSS type (critical, imposing, partial, undependable, shortsighted, and uncomforting social support respectively). Respondents were asked to rate the frequency of occurrence for each statement ranging on a scale from 1 to 6 for their coworker and supervisor. To reduce response burden, each item was presented once and rated side-by-side for coworkers and supervisor, respectively. An example item was: (my coworkers: …my supervisor: …) “Imply/implies that I’m incompetent when trying to help me complete a task” (see Gray (2018) p. 72–74 for a full overview of the UWSS question items). In addition, we created four items to measure poorly assigned social support, which was not included in the original scale as it applies only to supervisor support. An example item was: “My supervisor involves others in my tasks when their help is not really needed.” See Supplementary Table S1 for the Chronbach’s *α* values of the UWSS types in this study. Given the relatively recent development of the UWSS construct, we measured all dimensions. However, to ensure construct validity when testing the effects from supervisor and coworkers respectively, only the dimensions that demonstrated distinctiveness in factor analyses were retained for hypothesis testing.

#### 3.2.3 Dysfunctional social support (DSS)

The Bern Dysfunctional Social Support Scale (BDSSS) from Semmer et al. (2025) was adopted to measure DSS. The scale consists of eight items prompted by the following lead-in phrase: “How often does it happen that people at work help you in a difficult situation, but …” followed by a statement, for example: “combine this with reproaches.” Response alternatives ranged from 1 (almost never) to 7 (almost always). Chronbach’s *α* for DSS in this study was 0.87.

#### 3.2.4 Organization-based self-esteem (OBSE)

Organization-based self-esteem was measured using the 10-item scale adopted from Pierce et al. (1989). A sample item was: “I count around here.” Responses were assessed on a 5-point Likert scale ranging from strongly agree to strongly disagree.” Chronbach’s *α* for OBSE in this study was 0.89.

#### 3.2.5 Counterproductive work behaviors (CWB)

Counterproductive work behaviors were measured using Blau and Andersson’s (2005) instigated workplace incivility measure. The scale consists of seven items introduced by the following lead-in phrase: *“*How often have you exhibited the following behaviors in the past year to someone at work (e.g., co-worker, other employee, and supervisor)?” followed by a statement, for example: “put down others or were condescending to them in some way.” Response options ranged from 1 [hardly ever (once every few months or less)] to 4 [frequently (at least once a day)]. Chronbach’s α for CWB in this study was 0.82.

#### 3.2.6 Organizational frustration (OF)

Organizational frustration was measured using the same instrument employed by Gray et al. (2019), consisting of a modified 3-item version of the organizational frustration scale by Peters et al. (1980). A sample item was “Trying to get my job done is a frustrating experience.” Response alternatives ranged from 1 (strongly disagree) to 7 (strongly agree). Chronbach’s α for organizational frustration in this study was 0.80.

#### 3.2.7 Turnover intention (TI)

To measure turnover intention, we used the turnover intention scale from Cohen (1999). The scale consisted of 3 items with response alternatives ranging from 1 (strongly agree) to 5 (strongly disagree). A sample item was “I think a lot about leaving the organization.” Chronbach’s α for turnover intention in this study was 0.88.

### 3.3 Strategy of analysis

The analytical strategy consisted of four steps: (1) calculating descriptive statistics, (2) testing the factor structure of the measures, (3) hypothesis testing, and (4) calculating indirect effects to test mediation. Path models were used to test the study hypotheses. Descriptive statistical analyses were conducted with Jamovi v. 2.2.5. Path models were estimated using the lavaan package (Rosseel, 2012) in R v. 4.1.0.

First, descriptive statistics such as intercorrelations, means and standard deviations of study variables were calculated. Next, before probing the study hypotheses, we examined the structural validity of the UWSS scales, as these have not previously been assessed with separate coworker and supervisor measures simultaneously, nor used together with the DSS items.

To explore the factor structure of the items and subscales comprising the UWSS- instrument, we conducted an exploratory factor analysis (EFA), with maximum likelihood extraction and oblimin rotation. The EFA contained all of the items from the UWSS subscales, including both coworker and supervisor ratings, as well as the items from the DSS scale. UWSS subscales that contained items with substantial cross-loadings were subsequently omitted, and only scales showing clear loadings onto separate factors for coworkers/supervisor, respectively, were retained for further analysis. We confirmed the final measurement model of the measures by means of a confirmatory factor analysis, where items from the scales were used as indicators for each latent variable, respectively. The latent variables, and residuals from items that shared exact phrasing albeit with different referents (e.g., the same item referring to coworkers and supervisors), were free to correlate in the model (Cole and Maxwell, 2003). Model fit indices were used to evaluate fit (confirmatory fit index [CFI] and Tucker-Lewis Index [TLI] > 0.95, root mean square error of approximation [RMSEA] < 0.06, standard root mean square residual [SRMR] < 0.08, and the χ^2^- test of the model-implied covariance matrix) (Hu and Bentler, 1999).

In the third step, we estimated a path model where the UWSS and DSS scales were specified as predictors of organization-based self-esteem (OBSE), which in turn predicted the three outcome variables, counterproductive work behaviors (CWB), organizational frustration (OF), and turnover intention (TI).

Fourth, and finally, direct paths were added from the UWSS and DSS variables to the outcomes to assess the presence of partial mediation, and the indirect effects were calculated to test the final hypothesis. To assess the significance of the indirect effects, we applied the 95% percentile bootstrap method (5,000 draws), to construct confidence intervals that account for the non-normal nature of indirect effects.

## 4 Results

### 4.1 Descriptives

Means, standard deviations, Chronbach’s alpha values, and Pearson’s correlation coefficients of the study variables are depicted in Supplementary Table S1. All UWSS variables were moderately to strongly correlated with each other. DSS was also moderately to strongly correlated with all UWSS scales (|*r*|s ≥ 0.33, *p* < 0.001). For the UWSS variables, the coworker and supervisor subscales were correlated in similar directions with all outcome variables, albeit with different strength and significance levels. Generally, the relationships with OBSE, OF, and TI were stronger for the UWSS supervisor measures.

### 4.2 Exploring the factor structure

An exploratory factor analysis was conducted with all items from both the coworker and supervisor-rated UWSS subscales and DSS jointly to address the study’s first and second aim: (1) examining whether UWSS and DSS represent conceptually and empirically distinct constructs and (2) investigating whether employees perceive and receive UWSS differently from coworkers versus supervisors. The Keyser-Meyer-Olkin (KMO) measure of sampling adequacy was 0.85, and Bartlett’s test of sphericity was significant, *χ*^2^(1653) = 7,332, *p* < 0.001. The DSS items all loaded on a single factor (loadings ranged from 0.55–71), suggesting that DSS is empirically distinct from UWSS. For UWSS, only the shortsighted and partial support subscales loaded on two separate factors each, one for coworker ratings, and one for supervisor ratings (all loadings > 0.55). The items concerning critical support, undependable support, and uncomforting support loaded on single factors for each construct which included both the supervisor and coworker ratings, suggesting that it is not empirically meaningful to distinguish between supervisors and coworkers for these UWSS types. Item loadings from imposing support and poorly assigned support were inconsistent and scattered across factors.

Since the hypothesized model aims to compare coworker and supervisor sources of UWSS, we chose to omit subscales where coworker and supervisor ratings were not clearly empirically distinct to ensure an appropriate measurement model of the constructs of interest. Consequently, we only retained the partial and shortsighted unhelpful social support scales (from coworkers and supervisors) in the subsequent hypotheses testing, together with the DSS scale. To verify the factor structure after initial item evaluation and ensure that the constructs were suitable for inclusion in the subsequent path models, we conducted a confirmatory factor analysis. The confirmatory factor analysis on items of the four UWSS subscales; partial coworker support, partial supervisor support, shortsighted coworker support, shortsighted supervisor support, and dysfunctional social support, revealed an acceptable fit to the data: χ^2^(234) = 454, *p* < 0.001, CFI = 0.92, TLI = 0.91, SRMR = 0.06, RMSEA = 0.078 [0.067–0.089]. While some of the indices diverged slightly from the recommended cutoffs, it is important to note that Hu and Bentler’s (1999) guidelines were not intended as rigid thresholds. Rather, they state that CFI and TLI values should be close to 0.95. Complex models, with numerous latent variables and indicators, such as the present one, often produce somewhat lower CFI and TLI values, which do not necessarily indicate model misspecification (Cheung and Rensvold, 2002). In light of this, we did not consider the deviation substantial enough to preclude further hypotheses testing.

### 4.3 Hypothesis testing

To test the study’s hypotheses aligning with the third aim of this study, a path model was estimated where partial and shortsighted support (both from coworkers and supervisors) were added as predictors of OBSE. In turn, OBSE had paths to CWB, OF, and TI. The model revealed significant negative effects from OBSE to CWB (*β* = −0.24, *p* = 0.002), OF (β = −0.38, *p* < 0.001), and TI (β = −0.43, *p* < 0.001). OBSE was significantly negatively predicted by partial supervisor support (β = −0.36, *p* < 0.001), but not by the other UWSS subscales. When adding DSS as a predictor to the model, there were two significant paths to OBSE; one significant path from partial supervisor support (β = −0.31, *p* < 0.001), and one slightly stronger path from DSS (β = −0.39, *p* < 0.001). Consequently, we found no support for H1a concerning unhelpful coworker support, whereas H1b concerning unhelpful supervisor support was partially supported. Finally, H1c, H2, H3, and H4 were all supported.

### 4.4 Analysis of indirect effects

**Figure 2:**
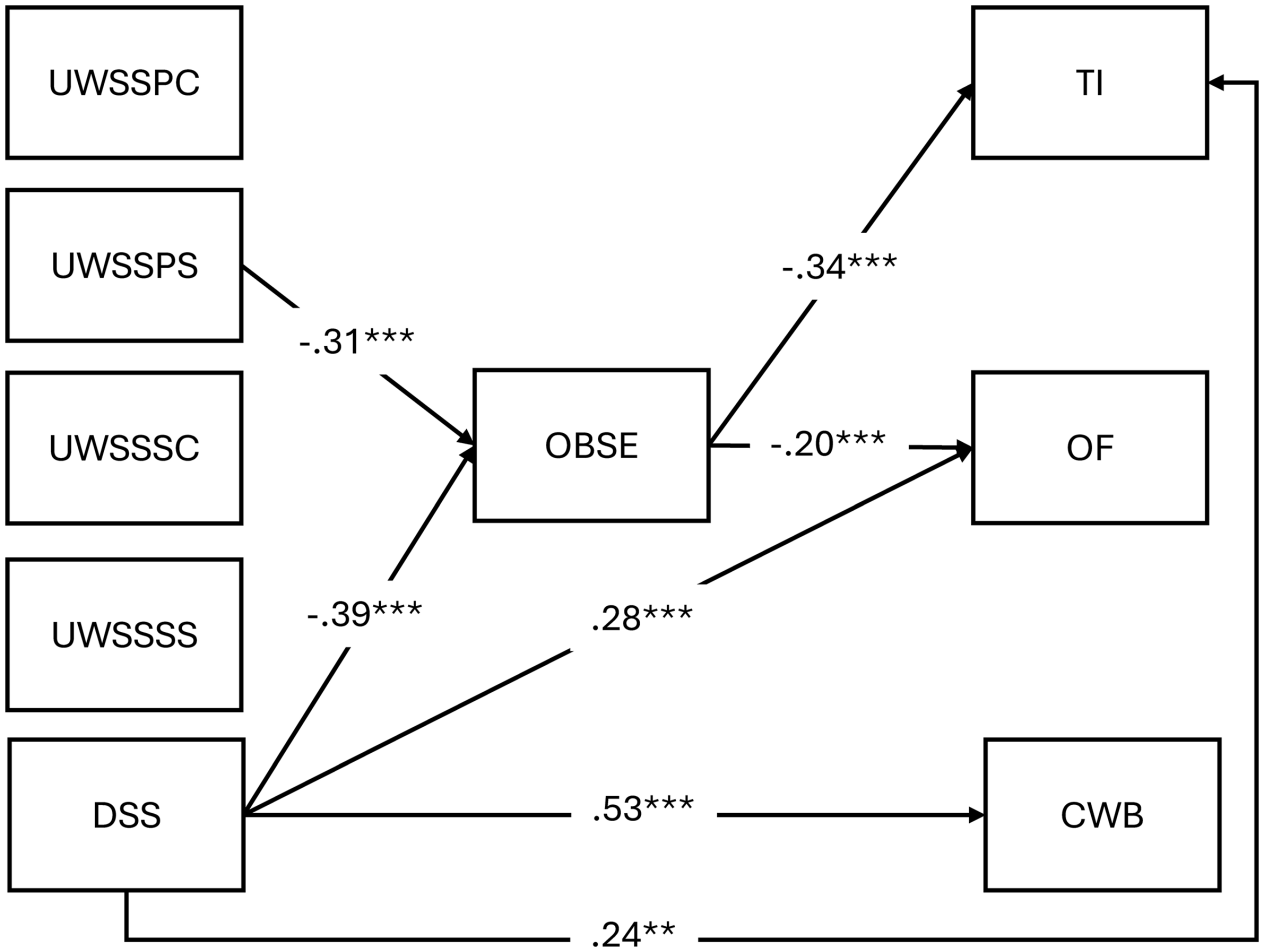
Path analysis results.

To test the final hypotheses H5a and H5b, whether UWSS/DSS were indirectly related to the outcomes *via* OBSE, we first added direct paths from the UWSS and DSS variables to the outcomes before calculating the indirect effects. When calculating the indirect effects, we applied bootstrapped standard errors (5,000 draws) to evaluate their significance. The final model is depicted in Figure 2.

Adding direct paths revealed significant paths from DSS to CWB (β = 0.53, *p* < 0.001), OF (β = 0.28, *p* = 0.001), and TI (β = 0.25, *p* = 0.005), but none from partial supervisor support to the three outcomes. However, for DSS, the path between OBSE and CWB was not significant (β = −0.10, *p* = 0.212), suggesting a direct rather than indirect relationship between DSS and this outcome. The significant paths for partial supervisor support from OBSE to OF and TI remained, but were slightly weaker (β = −0.20, *p* = 0.012, and β = −0.34, *p* < 0.001, respectively). Consequently, we only calculated the indirect effects from partial supervisor support and DSS to OF and TI.

The indirect path from partial supervisor support to OF (*B* = 0.078, [0.001–0.174] *p* = 0.080) was not significant. Conversely, the indirect path from partial supervisor support to TI (*B* = 0.15, [0.038–0.278], *p* = 0.012) was significant. Similarly, the indirect path from DSS to OF (*B* = 0.11, [0.005–0.235], *p* = 0.053) was just over the threshold for statistical significance, whereas the path to TI (*B* = 0.23, [0.090–0.382], *p* = 0.003) was significant. Accordingly, the role of OBSE as a link was only supported in the relationship between partial supervisor support/DSS and TI, showing partial support for H5a and H5b.

In conclusion, H1a was not supported, showing limited significance of UWSS from coworkers. H1b was partially supported, as of all UWSS types measuring supervisor support, only partial supervisor support was significant in our model. While there was support for H1c, H2 and H4, in line with the hypothesized model, there was only limited support for H5a and H5b, concerning indirect effects *via* OBSE. In addition, this was only the case for partial supervisor support, alongside DSS. Overall, based on these results it can be concluded that compared to UWSS, DSS showed more consistent relationships in our model concerning both direct and indirect effects.

## 5 Discussion

This study aimed to advance understanding of how unhelpful support operates in the workplace, particularly by clarifying the distinction between UWSS and DSS, examining whether associations differ per support source provider, and testing a model that includes OBSE as a mediator. The results suggest that DSS, which captures more subtle and low-threshold behaviors, represents a more consistent predictor of outcomes than UWSS in our model, and that source-based differences may be more nuanced and type-specific than previously assumed. Finally, the findings indicate that OBSE might be more relevant for explaining withdrawal-related outcomes, such as turnover intentions, than for the emotional or behavioral outcomes adopted in our model.

The first aim of this study was to examine whether UWSS and DSS represent conceptually and empirically distinct constructs. The factor-analytic results support this distinction: DSS loaded on a single factor, and no UWSS items cross-loaded with it, indicating that DSS represents a distinct, though related, construct compared to UWSS. Moreover, DSS showed more consistent associations with the variables in our model than partial supervisor support, suggesting DSS might have stronger explanatory power over UWSS. One explanation for the comparatively stronger role of DSS might lie in the construct’s focus on subtle, lower-threshold behaviors. Such behaviors might occur more frequently and be more easily normalized in everyday work interactions, making them particularly salient sources of strain (Semmer et al., 2025). In contrast, UWSS items primarily reflect more explicit or extreme forms of unhelpful support, which might occur less frequently and therefore showed weaker associations in our sample. From this perspective, DSS might be better suited to capture small, recurring, everyday interpersonal stressors that gradually erode employees’ self-evaluations and behavioral responses. These findings align with arguments from Semmer et al. (2025) that subtle DSS acts are especially relevant for understanding everyday interpersonal stress processes at work. However, as only the empirically distinct UWSS subscales were retained, whereas DSS was analyzed in its complete unidimensional form, comparisons between the two constructs should be interpreted with some caution. This selective retention of UWSS dimensions may partially explain why DSS emerged as a more consistent predictor of outcomes.

The second aim of the study was to investigate whether employees perceive and receive UWSS differently from coworkers versus supervisors. The factor-analytic results indicate that not all UWSS types clearly differentiate between coworker and supervisor support sources. Of the seven UWSS types included in our scale, only shortsighted support and partial support loaded on two distinct factors, one reflecting supervisor support and one reflecting coworker support. These findings suggest that UWSS provider differentiation may not always be meaningful, or that employees do not consistently distinguish between coworker and supervisor support when evaluating unhelpful interactions. In addition, item loadings for imposing support and poorly assigned support were inconsistent and scattered across factors, suggesting that not all theoretically proposed UWSS subtypes are empirically distinct and that these subscales show limited construct validity in the present study.

Remarkably, partial supervisor support was the only UWSS type that showed a significant relationship with OBSE in our hypothesized model. This raises the question why this specific form of UWSS, and specifically when originating from supervisors rather than coworkers, relates to OBSE. Partial support is defined as “social support that does not benefit the recipient because it is incomplete, imprecise, or unclear” (Gray, 2018, p. 62). Such ambiguous and fragmented help might implicitly signal that the support receiver is expected to “fill in the gaps” themselves, thereby placing responsibility or performance expectations without sufficient guidance or validation. When partial support is provided by supervisors, whose formal role includes providing support and who are central to performance appraisal and career advancement, it might be interpreted as a subtle signal of insufficient recognition. In turn, this ambiguity might undermine the support receiver’s confidence in their abilities, the legitimacy of their needs, worthiness of full support, or their perceived organizational worth, negatively affecting OBSE. Within the SOS theory, social self-esteem reflects the extent to which individuals feel esteemed, acknowledged, and appreciated by significant others (Semmer et al., 2019). Because supervisors have formal authority and are commonly perceived as representatives of the organization, they might therefore be considered a more salient “significant other”, and partial support from them might be interpreted as a signal about one’s standing and value within the organization, affecting OBSE. At the same time, the limited differentiation between coworker and supervisor UWSS types observed in the present study suggests that source effects might be specific to certain forms of UWSS rather than generalizable across all UWSS types. In our results, only partial and shortsighted support consistently separated by provider, whereas other dimensions failed to do so. This pattern implies that employees may not interpret most forms of unhelpful support differently depending on whether it comes from a coworker or a supervisor. Thus, the relevance of the provider may be situational and contingent on the nature of the unhelpful interaction rather than representing a broad or stable distinction across all UWSS domains, which can be important to consider in future studies where unhelpful support from supervisors and coworkers is investigated.

The third aim was to test organization-based self-esteem (OBSE) as a link between UWSS and relevant outcomes, such as organizational frustration (OF), counterproductive work behavior (CWB), and turnover intentions (TI). In the present study, OBSE showed an indirect association between partial supervisor support and turnover intentions, consistent with complete mediation. OBSE also displayed an indirect association between DSS and turnover intentions, although DSS continued to show a significant direct association with TI. In contrast, OBSE did not exhibit indirect associations linking partial supervisor support or DSS with CWB or OF. Instead, DSS showed direct associations with both CWB and OF, whereas partial supervisor support showed neither direct nor indirect effects with these outcomes. These findings diverge from the hypothesized model and from parts of prior UWSS research that reported associations with CWB and OF (Gray, 2018; Hughes et al., 2022). Additionally, this also raises questions about a central assumption of Stress-as-Offence-to-Self (SOS) theory. SOS theory proposes that effects of unhelpful support operate through threats to the social self-esteem (SAD process) (Semmer et al., 2019). However, the theory does not provide a specific operationalization of this mechanism. Therefore, OBSE was used as a context-specific operationalization of social self-esteem within organizational settings. In the present study, this mechanism was supported for the relationship between partial supervisor support/DSS and TI, as this association was mediated by OBSE. In contrast, DSS also showed direct associations with CWB, OF and TI independent of OBSE, suggesting that different forms of unhelpful support operate through distinct mechanisms and that SOS theory might not apply for all forms of unhelpful help.

OBSE reflects the extent to which employees feel valued, acknowledged, and accepted within their organization (Pierce et al., 1989), and therefore might lend itself particularly well for understanding withdrawal-related outcomes such as TI. When partial supervisor support/DSS negatively affect OBSE, thinking about leaving the organization might become a plausible coping response. That TI, arguably the most distal outcome in our model, was the only variable showing a significant indirect effect could suggest that OBSE primarily captures more enduring, organization-focused self-evaluative processes rather than immediate affective or behavioral reactions, or that the mechanisms in SOS theory might not be fully captured by OBSE alone and that additional processes beyond OBSE could be relevant in explaining how unhelpful support relates to employee outcomes.

The direct relationships observed between DSS and CWB, OF, and (partially) TI suggest that some consequences of DSS might arise through more immediate interpersonal mechanisms that bypass OBSE. For instance, social exchange theory by Blau (1964) posits that people respond to the treatment they receive with corresponding attitudes and behaviors over time (Cook and Rice, 2006). From a social exchange perspective, DSS might operate primarily through reciprocal exchange processes, in which responses are enacted implicitly over time without requiring reflective self-evaluations through OBSE. Accordingly, repeated exposure to subtle negative or dismissive support might therefore normalize or legitimize CWB through reciprocity. Given that the CWB measure in the present study focused on interpersonal behaviors, responses might reflect direct activation of reciprocity or retaliation norms rather than intrapersonal reflective evaluations of one’s organizational belonging. In this sense, behavioral reactions such as CWB might occur more proximally and interpersonally, whereas self-evaluative processes captured by OBSE might be relevant for explaining more distal, withdrawal-oriented outcomes such as TI.

To conclude, addressing the study’s overarching aim of investigating how unhelpful support is manifested in the workplace by studying the distinctiveness of UWSS and DSS as related constructs, diving into perceptions of provider source (coworkers versus supervisors), and seeing how UWSS/DSS relate to OBSE, CWB, and TI, the findings suggest a nuanced picture. OBSE appears to play a more limited role than anticipated, primarily linking partial supervisor support and DSS to withdrawal-oriented outcomes like TI, whereas CWB and OF seem to emerge through more immediate interpersonal processes as reflected in their direct association with DSS. This pattern only partially aligns with SOS theory, which proposes that stressors (such as UWSS or DSS) threaten one’s (social) self-esteem which elicits strain reactions (Semmer et al., 2019). One possible explanation is that OBSE, as an organization-specific operationalization of social self- esteem, may not fully capture the broader social self-esteem threats as emphasized in SOS theory. At the same time, this study is among the first to directly test the mechanism proposed within the SOS framework. Rather than contradicting SOS theory, the present findings suggest that its proposed mechanisms may be outcome-specific, highlighting the importance of considering both the outcome type and the timing through which strain responses develop when examining UWSS and DSS.

### 5.1 Limitations and future research directions

Several limitations of the present study should be acknowledged. First, the use of convenience sampling and the relatively small sample size might limit the generalizability of the findings. Reduced statistical power is particularly relevant for mediation models, which typically require larger samples to reliably detect indirect effects. Consequently, the present study might have been susceptible to Type II errors, such that some effects, particularly indirect relationships, might have remained undetected (Morling et al., 2018). It should also be noted that the cross-sectional nature of the data prevents establishing temporal ordering among the variables, limiting the conclusions of the present study to indirect associations rather than mediation. Nevertheless, identifying possible indirect associations can be informative for future studies where mediating mechanisms are explored with temporal separation. Future studies could draw on larger samples and use longitudinal designs to explore the stability of the factor solution and the indirect effects indicated in the present study, to assess the consistency of the findings. In addition, the reliance on self-reported survey data raises the possibility of common method variance, which might have influenced the observed relationships (Podsakoff et al., 2003).

Second, coworker UWSS items referred to coworkers in general, whereas supervisor UWSS items referred to a single, clearly identifiable individual. Accordingly, coworker UWSS was assessed at the group level, whereas supervisor UWSS was assessed at the individual level. This discrepancy might have introduced ambiguity and reduced reliability in the coworker measure, which could have made effects more difficult to detect and complicated comparisons between coworker and supervisor support. Future research could adjust the question items so that both groups are measured at the same level. This could contribute to both UWSS and USL literature.

Third, OBSE was used as the operationalization of social self-esteem to test mediation effects as proposed in SOS theory. While OBSE captures organization-based evaluations of self-worth and sense of belonging, the construct might be too organization-specific to reflect broader or relational forms of social self-esteem emphasized in the SAD processes of SOS theory. This measurement choice could explain why indirect effects were limited to TI. Future research might therefore consider alternative measures of social self-esteem that extend beyond organization-based evaluations, as these could be more sensitive mediators for outcomes where OBSE showed limited effects.

The study also highlights issues related to the measurement and conceptualization of UWSS types and provider differentiation. Imposing and poorly assigned support showed inconsistent factor loadings, raising questions about their conceptual clarity and measurement validity. Future studies might therefore consider omitting these UWSS types or refine their operationalization to ensure clearer construct validity. The finding that only partial and shortsighted support differentiated clearly between coworker and supervisor sources suggests that provider distinction might not be equally relevant across all UWSS types. Nevertheless, because provider differences emerged for partial support, future research could explore provider differences in the DSS measure. Examining whether source differences exist in subtle, low- threshold dysfunctional support behaviors could provide a valuable extension of the DSS literature, which was not addressed in the present study.

Finally, drawing on social exchange theory, future studies could investigate whether exposure to DSS increases the likelihood of engaging in similar DSS behaviors towards others. Exploring such imitative or reciprocal responses could advance understanding of the underlying mechanisms of UWSS and DSS and how they spread within work environments.

### 5.2 Implications

The findings of the present study have both theoretical and practical implications. Taken together, the results of this study point to three interrelated insights: (1) DSS and UWSS are distinct constructs, and DSS was a comparatively more consistent predictor of outcomes than UWSS, (2) support provider differences appear limited and type-specific, and (3) OBSE appears to be particularly relevant for explaining withdrawal-related outcomes such as turnover intentions in our model. As introduced in the literature, support is not inherently beneficial, its effectiveness depends on the way it is delivered. This reinforces the notion that distinguishing between different forms of unhelpful support is critical, as subtle and low-threshold behaviors captured by DSS might play a more central role in everyday workplace stress processes than more explicit forms captured by UWSS. These findings highlight the relevance of subtle, low-threshold forms of unhelpful help which might be easily normalized in everyday workplace interactions yet have meaningful consequences. However, given the cross-sectional design, the implications should be interpreted with caution and not as causal recommendations.

From a theoretical perspective, the stronger effects of DSS suggest that UWSS might insufficiently capture the full range of problematic support behaviors. While UWSS captures more explicit and clearly identifiable forms of unhelpful support, DSS focuses on more subtle, indirect, and ambiguous behaviors. Factor-analytic results further suggest that the current UWSS measure might require refinement, as several subscales showed weak or inconsistent factor structures, only partial supervisor support significantly correlated with the outcome variables, and few UWSS subscales meaningfully differentiated between coworker and supervisor sources. In this respect, DSS and UWSS are empirically distinct constructs, but DSS might outperform UWSS as an explanatory construct for understanding support-related strain processes in some contexts.

Practically, the results emphasize that organizations may consider not only whether support is provided, but also how it is delivered. Well-intentioned support that is partial, inattentive, or subtly unappreciative can elicit negative behavioral or withdrawal-related outcomes. Notably, the findings suggest that supervisor provided support might be particularly influential for OBSE, as partial supervisor support was the only UWSS type that significantly predicted OBSE. Moreover, partial supervisor support was strongly associated with TI indirectly via OBSE, highlighting OBSE as a potentially relevant mechanism for employee retention. Interventions aimed at reducing turnover could therefore possibly benefit from targeting supervisor behaviors that communicate recognition, completeness, and genuine engagement when providing support, although this interpretation should be treated with caution, given the cross-sectional design and the fact that only one UWSS subtype showed consistent effects.

Furthermore, the strong direct association between DSS and CWB, OF, and TI indicate that subtle forms of unhelpful help might provoke immediate (behavioral) reactions, rather than operating solely through self-evaluative mechanisms. Theoretically, this suggests that the relationship between DSS and outcomes might not operate through OBSE, implying that SOS theory could be less applicable for DSS than originally theorized, or that OBSE does not fully capture the mechanisms proposed in SOS theory and that alternative mechanisms might also be at play. Conversely, SOS theory appears relevant for UWSS, particularly partial supervisor support. Practically, these findings suggest that interventions should address not only overt unhelpful acts, but also subtle, normalized support behaviors that might be perceived as dismissive or unappreciative. Encouraging reflection on everyday support practices could help prevent the accumulation of small interpersonal stressors that contribute to dysfunctional behavior over time.

Taken together, these present findings suggest that organizations should move beyond a binary understanding of support as either present or absent. Effective support interventions should explicitly address the quality, completeness, and interpersonal understanding of support exchanges, with particular attention to supervisors and the subtle forms of support that are most easily overlooked yet potentially most harmful. Furthermore, DSS appears to represent a different form of unhelpful support than UWSS, underscoring the subtlety of these behaviors and their potentially substantial impact. In this respect, the findings suggest that understanding unhelpful support requires simultaneously considering its form (UWSS vs. DSS), its source, and the mechanisms through which it relates to outcomes.

## Data Availability

The datasets presented in this article are not openly available but the data set can be obtained from the corresponding author upon reasonable request. Requests to access the datasets should be directed to kristoffer.holm@mau.se.
