# Supplementary material for: Whose helping hurts? Source and construct differences in unhelpful workplace social support
Source: Front Psychol. 2026 May 13;17:1811297. doi: 10.3389/fpsyg.2026.1811297 (PMC13235982; doi:10.3389/fpsyg.2026.1811297)
Supplement: Supplementary file 1 [file Table_1.docx]

Table 1.

Intercorrelations, means, standard deviations and Chronbach’s alphas of study variables.

|  |  |  |  |  |  |  |  |  |  |  |  |  |  |  |  |  |  |  |  |  |  |  |  |  |  |  |  |  |  |  | |  | |  |  | |  | | |  |  | |  | |  |  |
| --- | --- | --- | --- | --- | --- | --- | --- | --- | --- | --- | --- | --- | --- | --- | --- | --- | --- | --- | --- | --- | --- | --- | --- | --- | --- | --- | --- | --- | --- | --- | --- | --- | --- | --- | --- | --- | --- | --- | --- | --- | --- | --- | --- | --- | --- | --- |
|  | | **UWSSCC** | | **UWSSCS** | | **UWSSIC** | | **UWSSIS** | | **UWSSPC** | | **UWSSPS** | | **UWSSUPC** | | **UWSSUPS** | | **UWSSSC** | | **UWSSSS** | | **UWSSUC** | | **UWSSUS** | | **UWSSPAS** | | **DSS** | | **OBSE** | | | | **CWB** | | | **OF** | | | | **TI** | | | |  |  |
| UWSSCC |  | — |  |  |  |  |  |  |  |  |  |  |  |  |  |  |  |  |  |  |  |  |  |  |  |  |  |  |  | |  | |  | | |  | |  |  | | |  | |  | |  |
| UWSSCS |  | .70 | *** | — |  |  |  |  |  |  |  |  |  |  |  |  |  |  |  |  |  |  |  |  |  |  |  |  |  | |  | |  | | |  | |  |  | | |  | |  | |  |
| UWSSIC |  | .55 | *** | .35 | *** | — |  |  |  |  |  |  |  |  |  |  |  |  |  |  |  |  |  |  |  |  |  |  |  | |  | |  | | |  | |  |  | | |  | |  | |  |
| UWSSIS |  | .42 | *** | .50 | *** | .57 | *** | — |  |  |  |  |  |  |  |  |  |  |  |  |  |  |  |  |  |  |  |  |  | |  | |  | | |  | |  |  | | |  | |  | |  |
| UWSSPC |  | .57 | *** | .36 | *** | .47 | *** | .33 | *** | — |  |  |  |  |  |  |  |  |  |  |  |  |  |  |  |  |  |  |  | |  | |  | | |  | |  |  | | |  | |  | |  |
| UWSSPS |  | .28 | *** | .50 | *** | .28 | *** | .46 | *** | .47 | *** | — |  |  |  |  |  |  |  |  |  |  |  |  |  |  |  |  |  | |  | |  | | |  | |  |  | | |  | |  | |  |
| UWSSUPC |  | .42 | *** | .41 | *** | .41 | *** | .41 | *** | .53 | *** | .31 | *** | — |  |  |  |  |  |  |  |  |  |  |  |  |  |  |  | |  | |  | | |  | |  |  | | |  | |  | |  |
| UWSSUPS |  | .28 | *** | .43 | *** | .36 | *** | .46 | *** | .35 | *** | .56 | *** | .58 | *** | — |  |  |  |  |  |  |  |  |  |  |  |  |  | |  | |  | | |  | |  |  | | |  | |  | |  |
| UWSSSC |  | .49 | *** | .39 | *** | .67 | *** | .39 | *** | .53 | *** | .35 | *** | .45 | *** | .45 | *** | — |  |  |  |  |  |  |  |  |  |  |  | |  | |  | | |  | |  |  | | |  | |  | |  |
| UWSSSS |  | .37 | *** | .48 | *** | .41 | *** | .56 | *** | .39 | *** | .52 | *** | .41 | *** | .52 | *** | .58 | *** | — |  |  |  |  |  |  |  |  |  | |  | |  | | |  | |  |  | | |  | |  | |  |
| UWSSUC |  | .53 | *** | .49 | *** | .46 | *** | .35 | *** | .58 | *** | .38 | *** | .51 | *** | .49 | *** | .56 | *** | .46 | *** | — |  |  |  |  |  |  |  | |  | |  | | |  | |  |  | | |  | |  | |  |
| UWSSUS |  | .32 | *** | .58 | *** | .25 | ** | .41 | *** | .30 | *** | .61 | *** | .33 | *** | .49 | *** | .34 | *** | .52 | *** | .64 | *** | — |  |  |  |  |  | |  | |  | | |  | |  |  | | |  | |  | |  |
| UWSSPAS |  | .35 | *** | .38 | *** | .38 | *** | .42 | *** | .33 | *** | .41 | *** | .42 | *** | .59 | *** | .38 | *** | .42 | *** | .48 | *** | .55 | *** | — |  |  |  | |  | |  | | |  | |  |  | | |  | |  | |  |
| DSS |  | .34 | *** | .39 | *** | .43 | *** | .47 | *** | .39 | *** | .38 | *** | .33 | *** | .38 | *** | .41 | *** | .45 | *** | .49 | *** | .42 | *** | .54 | *** | — |  | |  | |  | | |  | |  |  | | |  | |  | |  |
| OBSE |  | -.12 |  | -.23 | ** | -.05 |  | -.18 | * | -.17 | * | -.37 | *** | .04 |  | -.08 |  | -.06 |  | -.19 | * | -.18 | * | -.37 | *** | -.33 | *** | -.41 | *** | | — | |  | | |  | |  |  | | |  | |  | |  |
| CWB |  | .09 |  | .09 |  | .18 | * | .12 |  | .11 |  | .02 |  | .15 |  | .14 |  | .10 |  | .06 |  | .17 | * | .08 |  | .32 | *** | .47 | *** | | -.25 | | ** | | | — | |  |  | | |  | |  | |  |
| OF |  | .16 |  | .27 | *** | .17 | * | .24 | ** | .28 | *** | .33 | *** | .30 | *** | .29 | *** | .17 | * | .29 | *** | .19 | * | .23 | ** | .40 | *** | .44 | *** | | -.38 | | *** | | | .33 | | *** | — | | |  | |  | |  |
| TI |  | .16 | * | .29 | *** | .17 | * | .29 | *** | .05 |  | .21 | ** | .07 |  | .13 |  | .12 |  | .23 | ** | .20 | * | .36 | *** | .39 | *** | .38 | *** | | -.44 | | *** | | | .22 | | ** | .42 | | | *** | | — | |  |
| *M* (*SD*) |  | 1.35 | (.71) | 1.44 | (.88) | 1.65 | (.87) | 1.77 | (1.04) | 1.67 | (.94) | 2.07 | (1.21) | 1.63 | (.97) | 1.67 | (.97) | 1.43 | (.82) | 1.56 | (1.05) | 1.31 | (.73) | 1.50 | (.97) | 1.82 | (.95) | 2.64 | (1.03) | | 5.89 | | (.86) | | | 1.64 | | (.72) | 3.39 | | | (1.52) | | 2.94 | | (1.78) |
| α |  | .84 |  | .89 |  | .83 |  | .83 |  | .88 |  | .90 |  | .90 |  | .85 |  | .91 |  | .94 |  | .81 |  | .87 |  | .79 |  | .87 |  | | .89 | |  | | | .82 | |  | .80 | | |  | | .88 | |  |

 Note. * *p* < .05, ** *p* < .01, *** *p* < .001. UWSCC = critical support coworkers, UWSSCS = critical support supervisor, UWSSIC = imposing social support coworkers, UWSSIS = imposing social support supervisor, UWSSPC = partial social support coworkers, UWSSPS = partial support supervisor, UWSSUPC = undependable support coworkers, UWSSUPS = undependable support supervisor, UWSSSC = shortsighted support coworkers, UWSSSS = shortsighted support supervisor, UWSSUC = uncomforting support coworkers, UWSSUS = uncomforting support supervisor, UWSSPAS = poorly assigned support, DSS = Dysfunctional social support, OBSE = Organization-based self-esteem, CWB = counterproductive workplace behaviors, OF = organizational frustration, TI = Turnover Intentions.
